# Evidence for local dendritic cell activation in pulmonary sarcoidosis

**DOI:** 10.1186/1465-9921-13-33

**Published:** 2012-04-18

**Authors:** Bregje Ten Berge, Alex KleinJan, Femke Muskens, Hamida Hammad, Henk C Hoogsteden, Rudi W Hendriks, Bart N Lambrecht, Bernt Van den Blink

**Affiliations:** 1 Department of Pulmonary Medicine, Erasmus MC, Dr. Molewaterplein 50, 3015, GE Rotterdam, The Netherlands; 2 Department of Respiratory Diseases, Laboratory of Mucosal Immunology, MRBI, University Hospital Gent, De Pintelaan 185, B9000 Ghent, Belgium

**Keywords:** Sarcoidosis, Dendritic cells, Bronchoalveolar lavage, Granuloma, TNFα

## Abstract

**Background:**

Sarcoidosis is a granulomatous disease characterized by a seemingly exaggerated immune response against a difficult to discern antigen. Dendritic cells (DCs) are pivotal antigen presenting cells thought to play an important role in the pathogenesis. Paradoxically, decreased DC immune reactivity was reported in blood samples from pulmonary sarcoidosis patients. However, functional data on lung DCs in sarcoidosis are lacking. We hypothesized that at the site of disease DCs are mature, immunocompetent and involved in granuloma formation.

**Methods:**

We analyzed myeloid DCs (mDCs) and plasmacytoid DCs (pDCs) in broncho-alveolar lavage (BAL) and blood from newly diagnosed, untreated pulmonary sarcoidosis patients and healthy controls using 9-color flowcytometry. DCs, isolated from BAL using flowcytometric sorting (mDCs) or cultured from monocytes (mo-DCs), were functionally assessed in a mixed leukocyte reaction with naïve allogeneic CD4+ T cells. Using Immunohistochemistry, location and activation status of CD11c^+^DCs was assessed in mucosal airway biopsies.

**Results:**

mDCs in BAL, but not in blood, from sarcoidosis patients were increased in number when compared with mDCs from healthy controls. mDCs purified from BAL of sarcoidosis patients induced T cell proliferation and differentiation and did not show diminished immune reactivity. Mo-DCs from patients induced increased TNFα release in co-cultures with naïve allogeneic CD4^+ ^T cells. Finally, immunohistochemical analyses revealed increased numbers of mature CD86^+ ^DCs in granuloma-containing airway mucosal biopsies from sarcoidosis patients.

**Conclusion:**

Taken together, these finding implicate increased local DC activation in granuloma formation or maintenance in pulmonary sarcoidosis.

## Background

Sarcoidosis is a systemic disease characterized by the presence of noncaseating granulomas in involved organs, affecting the lung in more than 90% of patients [1,2]. The granulomatous reaction occurs in the absence of a clearly defined immunological target. However, a reaction to an unidentified antigen is suspected [3]. An antigen-driven pathogenesis is supported by disease-associated polymorphisms in genes encoding antigen recognizing or antigen presenting molecules such as Toll-like receptors and MHC class II [4]. Epidemiological and experimental data are suggestive of airborne or infectious antigens, in particular mycobacterial peptides, but attempts to link sarcoidosis to a causative pathogen are difficult and remain controversial [5-7]. Increased numbers of CD4^+ ^T cells in the broncho-alveolar lavage (BAL) fluid are a further hallmark of disease [3,4]. Increased proportions of oligoclonal CD4^+ ^T cells were found in the BAL from patients with sarcoidosis, consistent with a MHC-restricted antigen-driven process [8,9]. Both granuloma formation and T cell alveolitis have been characterized as Th-1 responses [3,4,10-12]. These data have led to the hypothesis that sarcoidosis emerges from an exaggerated Th1 immune response upon presentation of an unidentified antigen by an antigen presenting cell (APC).

Past studies on APCs involved in pulmonary sarcoidosis focused on alveolar macrophages [8,9,13]. However, in recent years it has become clear that dendritic cells (DCs) are the key APCs in the lung, responsible for presentation of antigen in draining lymph nodes, inducing T cell activation and proliferation [14,15]. Models of granulomatous disease in response to mycobacterial antigens showed that DCs contribute to granuloma formation [16-18]. We recently found that pulmonary granuloma formation is dependent on the presence of DCs and DC-induced T cell proliferation in draining lymph nodes [19]. These data suggest that DCs are pivotal mediators in the pathogenesis of sarcoidosis. Indeed, DCs were observed in skin, lymph node and lung lesions from sarcoidosis patients [14,20]. Lymph node granulomas contained many mature DCs expressing the lysosome-associated membrane glycoprotein DC-LAMP, which is induced upon DC maturation [21]. These DC-LAMP^+ ^DC were typically located in the lymphocyte layer of granulomas and adjacent to CD3^+ ^T cells, suggesting functional DC-T cell interaction [21]. In muscular sarcoidosis, recruitment of mDCs and upregulation of the CD40/CD40L system in affected muscles suggested that mDCs would be involved in granulomatous inflammation through antigen presentation in a Th1 immune milieu [22]. However, there is debate about the number and function of DCs in pulmonary sarcoidosis: numbers of myeloid DCs (mDCs) and plasmacytoid DCs (pDCs) in peripheral blood of pulmonary sarcoidosis patients were reported to be either normal or reduced [21,23]. On the other hand, proportions of pDC and mDC in the BAL of sarcoidosis patients were reported to be similar and increased, respectively, when compared with healthy controls [24]. Also, decreased proportions of BAL mDCs were found positive for CD83 and CD86, suggesting an immature phenotype of these cells [24,25]. Furthermore, peripheral blood mDCs and *in vitro *differentiated monocyte-derived DCs (mo-DCs) from sarcoidosis patients demonstrated either a decreased or a normal ability to stimulate T cells in co-culture experiments [23,26]. These data have led to the prevailing opinion that in pulmonary sarcoidosis, DCs are immature and anergic in the lung [25]. Thus, the exaggerated immune response in pulmonary sarcoidosis is paradoxically associated with DCs displaying diminished immunoreactivity. Studies into this area have been hampered by technical difficulties in isolating functionally active DCs with a high degree of purity from the site of active disease. It therefore remained unclear whether pulmonary DCs are functionally different in sarcoidosis.

In this study, we set out to investigate whether local DCs are functionally different in patients with pulmonary sarcoidosis, employing our recently developed cell sorting strategy to isolate functionally active DCs from BAL fluid [27]. We found that at the site of disease, more and mature DCs are present in association with granulomas. Furthermore, DCs from patients with sarcoidosis display intrinsic properties associated with induction of an exaggerated immune response.

## Materials and methods

### Patients and healthy control subjects

After informed consent, a total of 44 patients with sarcoidosis underwent fibre-optic bronchoscopy. The diagnosis of sarcoidosis was made according to the guidelines of the ATS/ERS/WASOG statement on sarcoidosis [2]. None of the patients were on systemic corticosteroid or immunosuppressive drugs one year previous to or at the time of diagnosis and sampling. Patient characteristics are shown in Table 1. 34 healthy controls (male/female ratio of 11/22, median age 22.5 (19-31), 3 smokers) underwent fibre-optic bronchoscopy with BAL (14 controls) or mucosal biopsies (20 controls, from a previous study). As a further control, BAL from three subjects, all smokers, who underwent bronchoscopy to exclude pulmonary sarcoidosis (a 41 yr old patient with uveitis, a 32 yr old patient with ocular vasculitis and a 50 year old patient with parotic enlargement), and that were found to be without sarcoidosis or signs of pulmonary disease at time of bronchoscopy or during 2 years of follow up, was used. The protocol was approved by the Medical Ethical Committee of the Erasmus MC in Rotterdam. In different experiments, samples from subsets of patients and controls were used due to limits in sample availability. When applicable, key characteristics of patient/control subsets are mentioned with each experiment.

**Table 1:** Patient characteristics

	Patients (n = 44)
Age, years	37.5 (18-67)

Sex, male/female	17/27

Smoking, yes/no	8/36

Stage, (I/II)	21/23

Diagnosis based on:	

Biopsy	17

CD4/8 ratio	27

CD4/8 ratio, mean	4.8 (1.2-10)

Extra-thoracic sarcoidosis (number of subjects)	Eye (12), Skin (10), Joints/Arthralgia (9)

Co-morbidities (number of subjects)	Cardiovascular, including hypertension (3), Diabetes Mellitus (2), Thyroid disease (2) Sickle-cell disease (1), Psoriasis (1), Migraine (2)

Data are presented as median (range), unless otherwise stated.

### Collection of BAL, peripheral blood cells and mucosal biopsies

BAL was performed with a flexible fibre-optic bronchoscope (Olympus) placed in the right middle lobe in wedge position. Four aliquots of 50 ml saline were instilled and subsequently gently aspirated. BAL fluid was collected in siliconized bottles to prevent cell adherence and kept at 4°C. BAL fluid was filtered through a 100 μm cell strainer (BD Biosciences) and centrifuged for 7 min at 450 g at 4°C. Supernatants were aliquoted for ELISA. A portion of BAL fluid cells were counted and used directly to assess DC number and phenotype. Remaining BAL cells were frozen in 1 ml RPMI 1640 (Gibco), 10% FCS, 10% DMSO in a cryovial using a 5100 Cryo 1°C Freezing Container (Nalgene) to -80°C and stored at -150°C. Upon thawing, macrophages were depleted before further flowcytometric analysis or sorting, as described previously [27]. Peripheral blood mononuclear cells (PBMCs) were collected as described before and used directly to assess DC number and phenotype or frozen as described above [27]. Mucosal biopsies were frozen in Tissue Tek, O.C.T. compound, (Sakura Finetek Europe) and stored at -80°C.

### Flow cytometric analysis and sorting

PBMCs, BAL cells or cultured DCs were incubated in FACS buffer (PBS supplemented with 0.25% BSA, 0.5 mM EDTA and 0.05% NaN3 and stained with the following antibodies: FITC-conjugated anti-CD3 (UCHT1), anti-CD14 (61D3), anti-CD56 (MEM188), anti-CD4 PE-Cy5 (RPA-T4), anti-CD123-PE (6H6), anti-FoxP3-APC, all from eBiosciences, FITC-conjugated anti-CD16 (3 G8), anti-CD19 (HIB19) and anti-CD25 (2A3), PE-conjugated or biotinylated anti-CD80 (L307.4), anti-CD86 (2331(FUN-1)) or CD45RO (UCHI-1), APC-conjugated anti-CD83 (HB15e) and anti-CD40 (5 C3), anti-CD11c PE-Cy5 (B-ly6) and anti-HLA-DR PE-Cy7 (L243), all from BD Biosciences, as well as anti-CD123-APC (AC145, Miltenyi), anti-CXCR3-PE (49801)(R&D) and anti-DC-LAMP-PE (104.G4) (Immunotech). 1% heat inactivated human serum was added to prevent non-specific antibody staining. Fixable Aqua Dead Cell Stain kit for 405 nm (Invitrogen, Molecular Probes) was used as a live/dead marker. Cells were measured on a LSRII Flowcytometer (BD Biosciences). pDCs and mDCs were recognized based on forward and side scatter characteristics and expression of different markers. A FITC-labeled lineage mix, containing antibodies against CD3, CD14, CD16, CD19 and CD56 was used. mDCs were alive, low autofluorescent, lineage mix negative, HLA-DR^+ ^cells, CD11c^+ ^cell, pDCs were CD11c^- ^but CD123^+^. Isolation of mDCs from thawed BAL cells was performed using a FACS ARIA (BD Biosciences with Diva software and for analysis FlowJo software (Tree Star Inc.) was used. In a previous study, we showed that mDCs do not show an altered phenotype or altered T cell activating capacity after a freeze-thaw cycle [27].

### Immunohistochemistry of airway mucosa biopsies

Immunohistochemistry was performed in a half automatic stainer (Sequenza) using primary monoclonal antibodies against human CD11c (SHCL-3; BD Biosciences) and CD86 (2331(FUN-1); BD Biosciences). DCs were identified based on morphology and CD11c positivity in a haematoxylin background staining, as previously described [28]. Control staining was performed with an irrelevant mAb of the same subclass. All biopsy sections were stained in one session to reduce inter-staining variation and analysed in a blinded fashion by two different researchers. Sections from lung mucosal biopsies fulfilled the following criteria: intact epithelium, a subepithelial mucosa of 100 μm depth and a good overall morphological quality.

### Functional testing of DCs in vitro

Isolated BAL mDCs were mixed with allogeneic naïve CD4^+ ^T cells isolated from a healthy donor as described previously [27] in a 1:20 ratio and cultured in RPMI 1640 medium at 37°C for 5 days. At day 5, a portion of the cells was harvested for flow cytometry measurements and to the remaining cells 3H-thymidine (0.5 μCi/well, Packard) was added and after 16 hours cell proliferation was determined by scintillation counting (Top count model B9912/VI, Packard Bioscience Benelux). In order to culture mo-DCs, monocytes were isolated from PBMCs using CD14 beads (Miltenyi), according to the manufacturer's instructions. Monocytes were then resuspended at 1 × 10^6 ^cells/ml in RPMI 1640 culture medium, supplemented with 10% FCS, 50 μg/ml gentamycin (Gibco), 1000 U/ml GM-CSF (Immuno Tools) and 200 U/ml IL-4 (R&D) and cultured at 37°C for 7 days [27]. Mo-DCs were co-cultured with allogeneic naïve CD4^+ ^T cells for 5 days. T cell labeling with carboxyfluorescein succinimidyl ester (CFSE)-was performed using standard procedures.

Cytokine measurements in supernatants of DC-T cell co-cultures was performed using the human in a *25*-*plex *Luminex assay cytokine and chemokine kit (*Invitrogen*, Carlsbad, CA) and run on a Luminex 100 System (Luminex Corporation, Austin, TX), according to the manufacturer's protocol.

### Statistical analysis

Results were compared using a two-tailed Mann Whitney *U *test. Differences were considered significant at P ≤ 0.05.

## Results

### BAL characteristics

In this study we investigated BAL, peripheral blood and mucosal biopsies from newly diagnosed sarcoidosis patients and healthy controls (see: Mat & Methods and Table 1 for patient characteristics). Sarcoidosis patients displayed increased BAL cellularity (median: 327 × 10^3 ^total cells/ml), compared to healthy controls (median: 118 × 10^3 ^total cells/ml, p = 0.06), in agreement with previous reports [27,29,30]. This increased cellularity in BAL was paralleled by increased proportions of lymphocytes and increased CD4/CD8 ratios (median: 4.8; range 1.2-22). BAL lymphocytes in sarcoidosis are reported to be highly positive for the Th1-specific chemokine receptor CXCR3 [31]. Accordingly, the closely related CXCR3 ligands CXCL9, also known as monokine induced by IFNγ- (MIG) and CXCL10, also known as IFNγ-induced protein of 10 kDa (IP-10), are elevated in BAL fluid [32]. When we measured the levels of these chemokines in BAL fluid in the same patients tested for mDC function, we found that they were elevated: for CXCL9 the median was 533 pg/ml (range 65-1397) in patients and 43 pg/ml (range 21-59; p = 0.0004) in healthy controls and for CXCL10 these values were 360 pg/ml (range 34-957) in patients and 0 pg/ml (0-96; p < 0.003) in controls.

### Increased mDCs in BAL and blood in sarcoidosis

**Figure 1:**
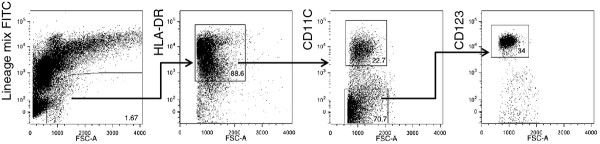
**Gating and sorting strategy for mDCs and pDCs**. Dead cells were excluded from the total fraction of the BAL cells using DAPI as a dead-live marker (not shown). The alive, low auto fluorescent, lineage mix (CD3-, CD14-, CD16-, CD19-, CD56-) FITC negative fraction was expressed in the Pe-Cy7 channel for HLA-DR expression. HLA-DR positive cells were subsequently expressed in the PE-Cy5 channel for CD11c expression. Cells positive for CD11c are mDCs. CD11c negative cells were expressed in APC for CD123: cells positive for CD123 are pDCs.

**Figure 2:**
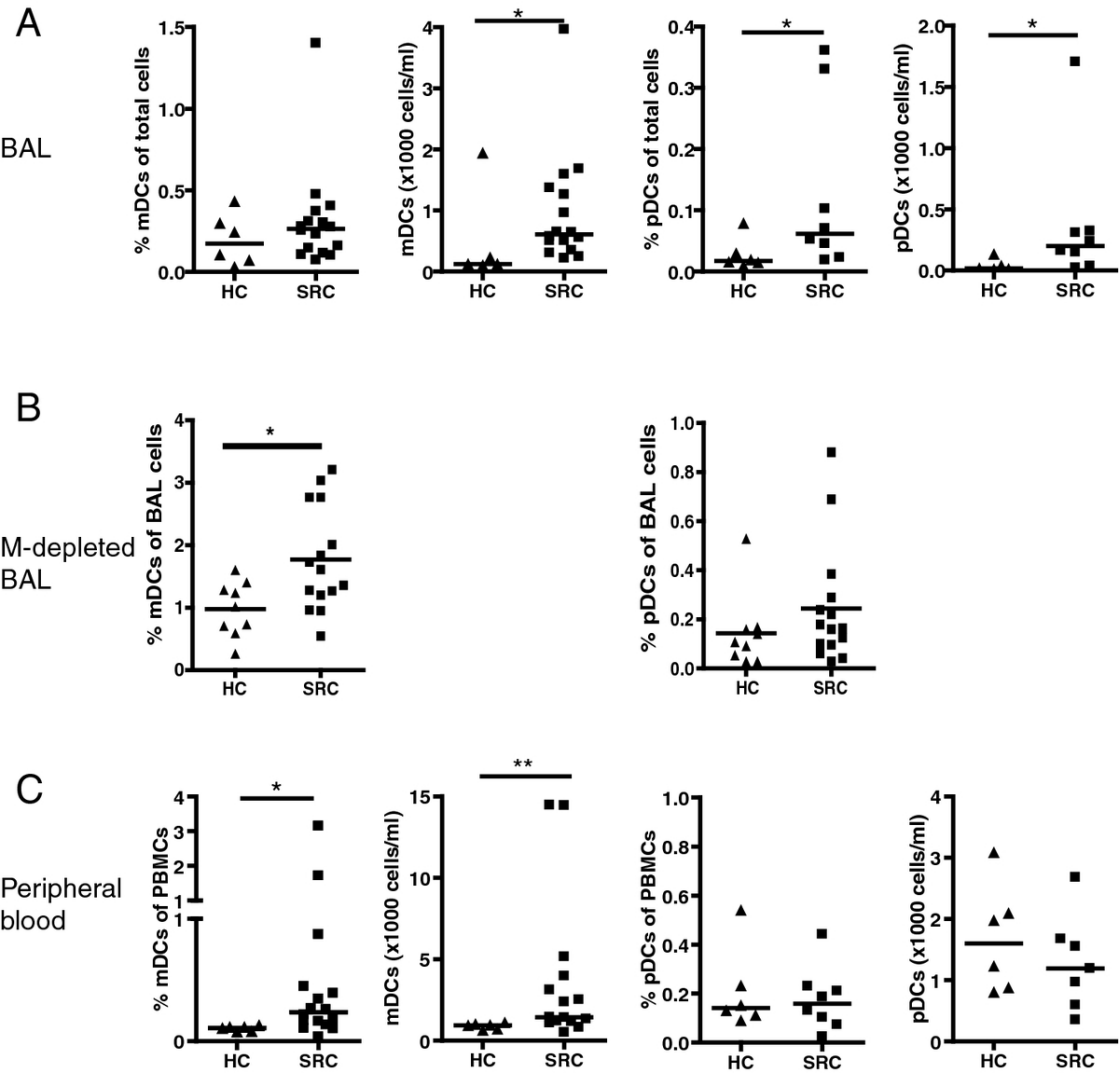
**Increased mDCs in BAL and blood in sarcoidosis**. Proportions and numbers of mDCs and pDCs in BAL, Macrophage (M-)depleted BAL and peripheral blood from sarcoidosis patients and healthy controls. Using 9-color flowcytometry, the proportion of mDCs (low autofluorescent, lineage mix negative, HLA-DR^+^, CD11c^+ ^cells) and pDCs (low autofluorescent, lineage mix negative, HLA-DR^+^, CD11c^-^, CD123^+ ^cells) was determined of total BAL cells (A), Macrophage (M-)depleted BAL cells (B), and PBMCs from peripheral blood (C). The number of mDCs and pDCs was calculated as a proportion of total counted BAL cells and PBMCs. Each symbol represents an individual patient or control; lines indicate medians. * p < 0.05; ** p < 0.01.

To investigate DCs in pulmonary sarcoidosis, we first analyzed the numbers of DC subsets in BAL and peripheral blood from patients (mDC (n = 16), pDC (n = 8)) and healthy controls (n = 6). The gating strategy used to identify mDCs and pDCs, shown in Figure 1, was previously published by us and others [27,33]. The proportions of HLA-DR^+^CD11c^+ ^mDCs in total BAL cells did not significantly differ between sarcoidosis patients and healthy controls (median: 0.26%, range: 0.07-1.4 and median: 0.17%, range: 0.03-0.43, respectively; Figure 2A), corroborating earlier studies [24]. However, the absolute numbers of mDCs were significantly elevated in patients (median 608 cells/ml, range: 217-3,965), when compared with healthy controls (median 123 cells/ml, range: 101-1,941, p = 0.05).

In the BAL from sarcoidosis patients the populations of interleukin-3 receptor (IL-3R)/CD123-expressing HLA-DR^+^CD11c^- ^pDCs were increased, both in proportions (median 0.06%, range 0.02-0.36, compared with healthy controls: median 0.03%, range 0.01-0.08, p = 0.03) and in absolute numbers (median: 201 cells/ml, range: 23-1740, compared with healthy controls: median 17 cells/ml, range 11-133, p = 0.02; Figure 2A).

In order to further validate these results, we additionally analyzed proportions of mDCs and pDCs in macrophage-depleted BAL from 15 patients and 9 controls. Median age of patients in this subgroup was 37 years (range: 23-69, 1 smoking) and controls 29 years (range: 21-50, 4 smoking). Controls consisted of 6 healthy controls and 3 individuals without pulmonary disease in whom there were a need to exclude sarcoidosis (see also Mat & Methods). After macrophage depletion, proportions of mDCs and pDCs were generally higher, as was to be expected. The proportions of mDCs in macrophage-depleted BAL were significantly elevated in patients (median 1.61%, range: 0.55-3.2), when compared with healthy controls (median 1.01%, range: 0.26-1.60, p = 0.025; Figure 2B). Proportions of pDCs in macrophage-depleted BAL tended to be higher in patients (median 0.16%, range: 0.03-0.88), when compared with healthy controls (median 0.11%, range: 0.03-0.52), but were not significantly different (Figure 2B).

In a blood sample, concurrently drawn from the patients who's BAL findings are reported in Figure 2A, we observed an increase in mDCs in sarcoidosis patients, both in proportions (median: 0.24%, range: 0.03-1.71, compared with healthy controls 0.11%, range 0.08-0.13, p = 0.01) and in numbers (median: 1,441/ml, range: 511-14,432, compared with healthy controls: median 933, range 638-1,102, p < 0.01; Figure 2C). In contrast, no differences were observed in pDC numbers or proportions between patients and controls (Figure 2C). Peripheral blood from sarcoidosis patients and healthy controls also did not differ in the total numbers of PBMCs per ml (data not shown). Overall, we did not observe an association between numbers of mDCs or pDCs in BAL or blood and disease stage, as classified by Scadding [34] (data not shown).

Next, we set out to investigate whether the increase in DCs in BAL of sarcoidosis patients was associated with enhanced maturation of DCs in BAL and blood. In peripheral blood the expression of the CD80, CD86, CD40, CD83 and CD208 maturation markers on mDCs or pDCs was low and did not differ between sarcoidosis patients and healthy control subjects (data not shown). When analyzing CD40, CD80 (B7-1), CD83, CD86 (B7-2), and CD208/DC-LAMP expression on mDCs and PDs from BAL we found considerable heterogeneity in marker expression in repeat experiments, precluding conclusions on differences in BAL mDC maturation between sarcoidosis patients and controls

In summary, in recently diagnosed sarcoidosis patients we found an increase in mDCs and pDCs in the BAL and mDCs in peripheral blood, irrespective of disease stage.

### BAL mDCs from sarcoidosis patients are immunocompetent

**Figure 3:**
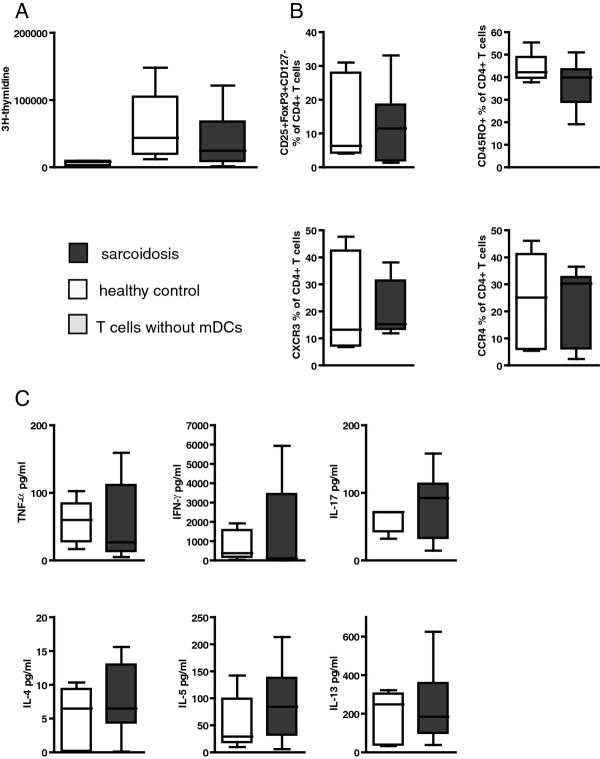
**BAL mDCs from sarcoidosis patients are immunocompetent in co-cultures with T cells**. Induction of T cell proliferation and differentiation by mDCs isolated from BAL from sarcoidosis patients. mDCs (defined as low autofluorescent, lineage mix negative, HLA-DR^+^, CD11c^+ ^cells), isolated from BAL from sarcoidosis patients and healthy controls, were tested in a MLR with naïve allogeneic CD4^+ ^T cells. (A) After 5 days of co-culture, T cell proliferation was measured using 3H-thymidine incorporation. (B) T cell differentiation was assessed using markers for Tregs (CD25+ Foxp3^+ ^CD127 ^low^, memory T cells (CD45RO^+^), Th1 (CXCR3^+^) and Th2 (CCR4^+^) cells. (C) Cytokines were measured in the supernatant of the co-culture using a bead-based cytokine kit. Data are presented as boxplots, whiskers indicate minimum and maximum of the data.

Next, we evaluated whether an increase in mDCs in the BAL from sarcoidosis patients was associated with either enhanced or diminished T cell activation, the latter of which has been suggested for blood-derived DCs in sarcoidosis [25]. We applied our previously developed flow cytometric strategy for the isolation of mDCs from BAL to sort mDCs from BAL of 9 sarcoidosis patients (1 smoker, median age 29, range 23-64) and 6 healthy controls (no smokers, median age 22.5, range 20-24). In a previous study we showed that using a newly developed technique mDCs can be isolated from BAL using flow cytometric sorting with a high degree of purity (approximately 94%, range 91,6-96,6) [27]. To test the capacity of these sorted mDCs to induce T cell proliferation and differentiation, we performed co-culture experiments with allogenic naïve CD4^+ ^T cells. Upon 5 days of co-culture, naïve CD4^+ ^T cells displayed clear proliferation, when compared with unstimulated CD4^+ ^T cells. No differences were found between co-cultures with BAL mDCs sorted from healthy controls or from sarcoidosis patients (as measured by 3H-thymidine incorporation; Figure 3A). In these co-culture experiments naïve CD4^+ ^T cells differentiated into CD25^+^CD127^low^Foxp3^+ ^regulatory T cells, CD45RO^+ ^memory T cells, CXCR3^+ ^Th1 or CCR4^+ ^Th2 cells, whereby we did not observe significant differences between co-cultures with mDCs from sarcoidosis patients and with mDCs from healthy controls (Figure 3B). Likewise, we found similar levels of cytokines, including TNFα, IFNγ, IL-4, IL-5, IL-13 and IL-17 (Figure 3C).

Taken together, these findings indicate that mDCs from the BAL from sarcoidosis patients were functional and did not display diminished immunoreactivity, when compared with mDCs from healthy controls.

### Monocyte-derived DCs from sarcoidosis patients induce increased TNFα expression

**Figure 4:**
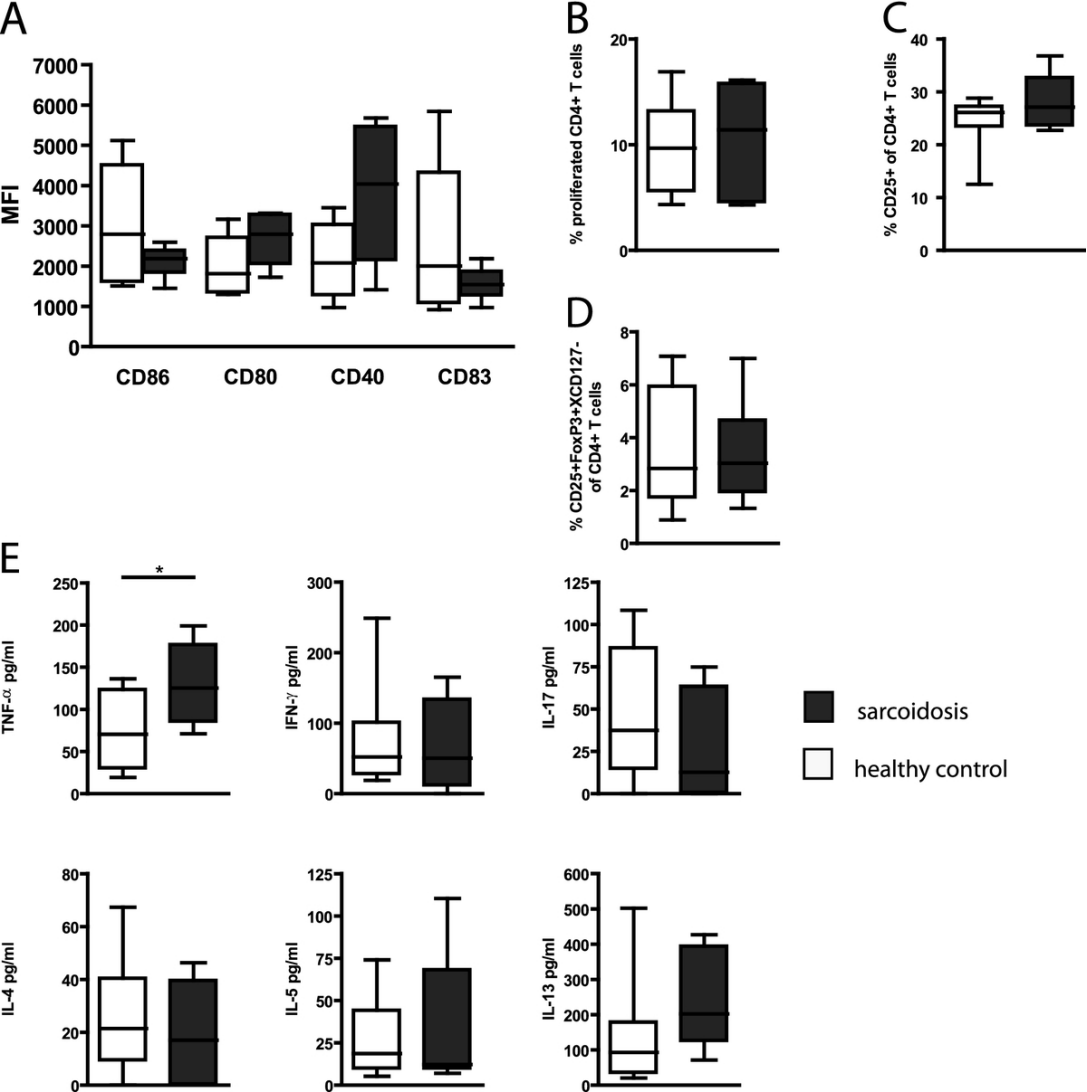
**Monocyte-derived DCs from sarcoidosis patients induce increased TNFα expression**. Activation and function of mo-DCs from sarcoidosis patients and healthy controls. (A) After culturing mo-DCs from monocytes, mean expression of DC maturation markers CD40, CD80, CD83 and CD86 was assessed by flowcytometry. (B) Mo-DC induced T cell proliferation was tested in a MLR after 5 days of co-culture with CFSE-labeled naïve CD4^+ ^T cells, using flowcytometry. (C, D) Mo-DC induced T-cell differentiation was measured by expression of CD25 (C) and Treg markers (CD25^+^Foxp3^+^CD127 ^low^)(D). (E) Cytokine production in DC-T cell co-cultures. Indicated cytokines were measured in the culture supernatants by Luminex. Data are presented as boxplots, whiskers indicate minimum and maximum of the data; * p ≤ 0.05.

To investigate whether intrinsic DC defects in sarcoidosis might lead to aberrant T cell responses independent of the lung microenvironment, we cultured monocyte-derived DCs (mo-DCs) from 10 sarcoidosis patients and 10 healthy controls. Expression of CD80, CD86, CD40 and CD83 was comparable in the two groups (Figure 4A). When mo-DC function was tested in co-cultures with allogeneic naïve CD4^+ ^T cells, mo-DCs from sarcoidosis patients and healthy controls did not manifest differences in their capacity to induce T cell proliferation (Figure 4B), T cell activation as assessed by membrane expression of the IL-2R/CD25 (Figure 4C), or regulatory T cell differentiation (Figure 4D). When cytokine production was measured by Luminex bead assays, we observed increased TNFα expression in co-cultures of mo-DCs from sarcoidosis patients, when compared with co-cultures of mo-DC from healthy controls (Figure 4E). Other cytokines tested, including IFNγ, IL-17, IL-4, IL-5 and IL-13 were not different between the two groups of cultures.

Taken together, these data show that DCs from sarcoidosis patients examined outside of the disease microenvironment were not intrinsically more mature, but do show an increased propensity to induce the production of TNFα, a cytokine pivotal in sarcoidosis pathogenesis.

### Increased numbers of mature CD86^+ ^DCs surrounding granulomas

**Figure 5:**
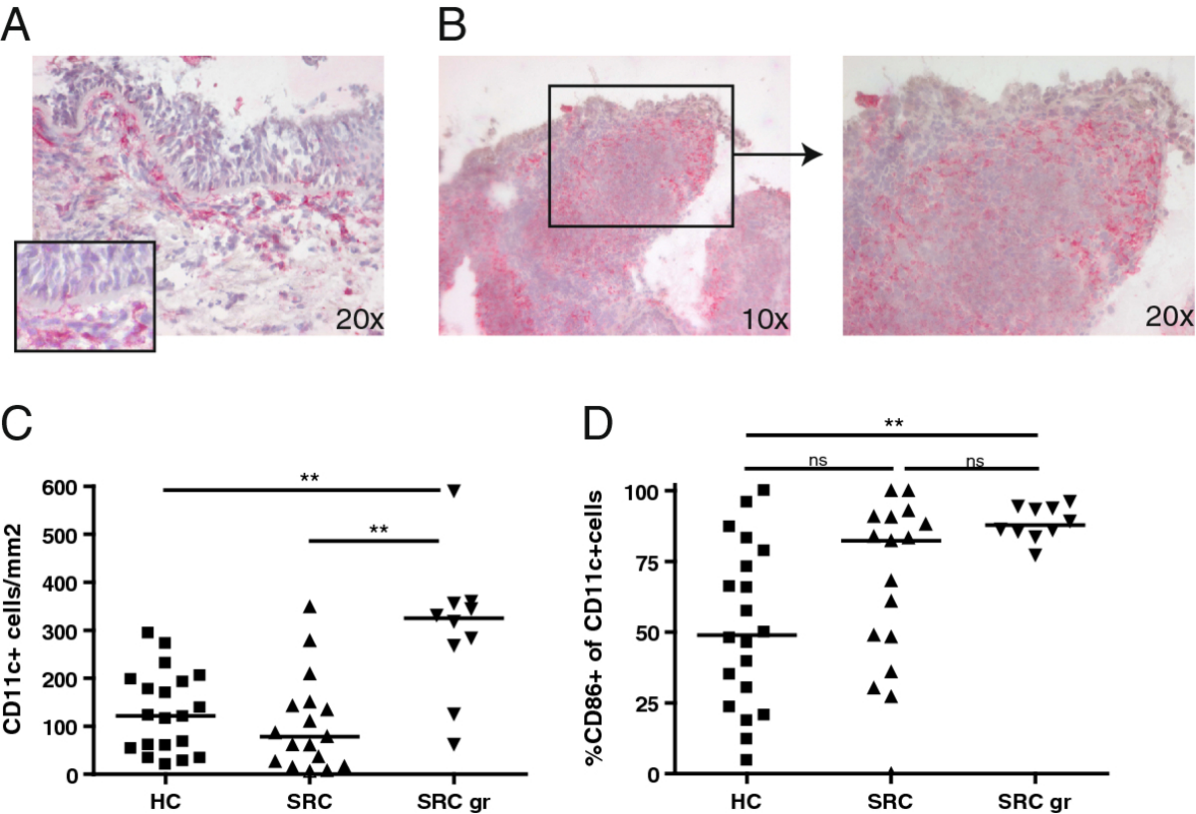
**Increased numbers of mature CD86^+ ^DCs surrounding granulomas in sarcoidosis**. Location, number and activation of DCs in mucosal airway biopsies. (A) CD11c staining on a frozen section from a mucosal airway biopsy from a healthy control, showing DCs in the subepithelial layer. Characteristic dendritic cell morphology was observed with dendrites crossing the basement membrane (*insert*). (B) In mucosal biopsies from sarcoidosis patients DCs were observed surrounding the granuloma. (C) Quantification of the numbers of CD11c^+ ^cells in lamina propria in biopsies from healthy controls (HC), sarcoidosis patients without (SRC) and with granuloma (SRC gr), bars indicate median values, ** p < 0.0015. (D) Proportions of CD11c^+ ^cells co-expressing CD86 in biopsies from healthy controls (HC), sarcoidosis patients without (SRC) and with granuloma (SRC gr). Each symbol represents and individual patient or control. Lines indicate median value, ** p < 0.0015 (D).

Finally, we determined the numbers, location and activation status of DCs in the airways in mucosal airway biopsies from 26 sarcoidosis patients and 20 healthy controls. Identification of DCs in biopsy material can be hampered by the absence of one marker unique for DCs; CD11c can be expressed on monocytes, macrophages and neutrophils as well. However, in combination with morphological features tissue DCs can be identified with confidence in histological sections, as we have previously shown [28].Cells positive for CD11c showing a characteristic DC morphology were found in the subepithelial layer of the airways in healthy subjects, as reported previously [35] (Figure 5A). Ten out of 26 mucosal biopsies from sarcoidosis patients contained clear granulomas, and in those CD11c^+ ^DCs were seen predominantly surrounding the granulomas, confirming earlier studies [21] (Figure 5B). We also observed weak CD11c expression in the center of granulomas, perhaps reflecting CD11c expression on myeloid derived epithelioid histiocytes or interdigitating dendrites. Granuloma-containing biopsies displayed significantly increased CD11c^+ ^cell numbers, when compared with either healthy controls (p = 0.0011) or non-granulomatous biopsies (p = 0.0014) (Figure 5C).

To assess the maturation state of the mucosal DCs, double stainings were performed for CD11c and CD86. In granuloma-containing biopsies, the proportions of CD11c^+ ^cells co-expressing CD86 was significantly increased (median 88%, range 77-96%), when compared with healthy controls (median 50%, range 4-95) (p = 0.0012), but not significantly different from values in non-granulomatous biopsies from patients (median 82%, range 0-100%). The proportion of CD86^+ ^DCs tended to be higher in non-granulomatous biopsies, compared with healthy controls, but differences did not reach significance, probably related to the variation of CD86^+ ^expression in healthy individuals.

Taken together, these results show that granuloma formation was associated with an increase in number and maturation status of DCs, providing evidence for the involvement of DCs in granuloma formation or maintenance in sarcoidosis.

## Discussion

DCs are pivotal antigen presenting cells and the prime suspects for initiating granuloma formation and T cell alveolitis characteristic for pulmonary sarcoidosis. Paradoxically, local DCs have been suggested to be phenotypically and functionally immature [25]. In contrast, we provide in this report the involvement of mature, functional DCs in pulmonary sarcoidosis. First, pulmonary sarcoidosis patients have increased numbers of mDCs in BAL, granuloma containing mucosal biopsies and blood. Second, mDCs in granuloma-containing biopsies show increased expression of a maturation marker. Third, mDCs from BAL are very well capable of inducing T cell proliferation and differentiation and show no signs of anergy. Finally, mo-DCs from sarcoidosis patients induce more TNFα in co-cultures with allogeneic CD4^+ ^T cells, compared to mo-DCs from healthy controls. Taken together these results indicate that pulmonary sarcoidosis is associated with increased numbers of mature, functionally competent DCs that intrinsically induce increased levels of a central mediator in sarcoidosis, TNFα.

Previous studies and ours report different results in numbers of mDCs in blood or BAL from sarcoidosis patients [21,23,24]. These differences may reflect variations in obtaining (e.g. volume of total BAL fluid), isolating (enriched cell populations vs. flow cytometric sorting), calculating (number per ml vs. proportions) and markers used for defining DC subsets. E.g we found increased proportions and numbers of mDCs in blood from sarcoidosis patients compared to healthy controls by calculating mDCs as the proportion of PBMCs from the same sample, while others reported (similar) numbers of mDCs as a proportion of total leucocytes, obtained by lysis of red blood cells, and calculated from a concurrent drawn blood sample [23], or even decreased numbers of mDCs calculated by using proportions of a DC-enriched cell population [21]. We performed BAL with 200 ml saline, compared to e.g. 100 ml in other reports [24]. It is conceivable that an increase in BAL fluid amount reduces the relative contribution of the larger conducting airways to the retrieved amount of fluid, thus increasing the cellular yield from the alveolar compartment, increasing the yield of DCs.

To the best of our knowledge we report for the first time functional tests on mDCs from the site of disease in pulmonary sarcoidosis. DCs isolated from BAL from sarcoidosis patients did not induce enhanced T cell proliferation, a skewed T cell differentiation or significant differences in the induction of several cytokines in a MLR with allogeneic naïve CD4+ T cells, compared to controls. Several explanations related to the micro-environment of the lung where DC-T cell interactions take place, are possible: First, DCs from BAL from sarcoidosis patients may have already interacted with T cells, and are beyond their functional maximum upon isolation from BAL. This is supported by studies showing that after initial exposure to a stimulus, DCs produce IL-12, important for inducing T cell proliferation, for a limited period of 10-18 hours [36]. Second, it is perhaps the sheer number of DCs and not the maturation status that determines the *in vivo *outcome of T cell proliferation in sarcoidosis. Although the number of DCs in BAL is in general very low, DCs in the BAL are thought to reflect only a small percentage of pulmonary DCs [37]. We found increased numbers of mDCs per ml of BAL in sarcoidosis patients, perhaps indicating increased numbers of interstitial DCs that may travel to the draining lymph node for antigen presentation. Future investigations in the lymph node compartment may shed light on this issue. Third, perhaps an intrinsic T cell factor is (additionally) required to induce the exaggerated T cell response observed in sarcoidosis. Interestingly, a single nucleotide polymorphism (SNP) in the IL-23 receptor was recently associated with sarcoidosis [38]. IL-23 is a cytokine that is essential for the induction of IL-17 producing CD4^+ ^T cells (Th17 cells) that were recently associated with granuloma formation in sarcoidosis by us and others [38,39]. Interestingly, we did observe a modest but not significant increase in IL-17 production in the MLR supernatant of sarcoidosis patients. Finally, influx of CD4^+ ^T cells into the bronchoalveolar space may primarily be determined by chemotactic factors. Our data on enhanced levels of MIG and IP10 in BAL, well-known chemotactic factors for Th1 cells, support this notion and confirm earlier reports [32,40].

TNFα is a pivotal mediator of granuloma formation and maintenance, and is thought to play an important role in sarcoidosis pathogenesis [41]. Indeed, enhanced TNFα secretion by BAL macro-phages is observed in sarcoidosis [3]. In addition, TNFα is also expressed by Th1 and Th17 cells and both T helper cell subsets are likely involved in sarcoidosis pathogenesis [38]. Polymorphisms in the *TNFα *locus were associated with sarcoidosis phenotype and prognosis and have been linked to altered TNFα expression [42,43]. Importantly, TNFα is an essential target for treatment [44] and we found that mo-DCs from patients with sarcoidosis, not influenced by the micro-environment of the lung, induced increased TNFα release upon interaction with naïve CD4^+ ^T cells when compared to controls. Our results indicate that DCs are intrinsically different in sarcoidosis patients. Nevertheless, we found that mo-DCs from sarcoidosis patients were equally capable of inducing proliferation and differentiation in allogeneic naïve T cells, compared to healthy controls. In the current study we did not explore whether mo-DCs from sarcoidosis patients lead to skewing of T cell differentiation towards a Th1/Th17 phenotype compared to controls, and this remains to be investigated. In co-cultures with PBMCs, mo-DCs were previously reported to demonstrate a reduced capacity to induce T cell proliferation (26). However, it is conceivable that other cells in the PBMC fraction, e.g. regulatory T cells (Tregs), influenced T cell proliferation. Indeed, we found increase numbers of Tregs in the PBMC fraction from sarcoidosis patients' blood (unpublished observations).

Granulomas are thought to arise upon failure of the immune system to clear invading pathogens. Earlier reports on diminished DC maturation and immune reactivity led to the notion that DC anergy may contribute to granuloma formation in sarcoidosis, e.g. due to diminished antigen presenting capabilities or ineffective induction of a T helper cell response [25]. However, we found that both mDCs in BAL and in granuloma-containing airway biopsies were increased in number and had enhanced expression of maturation marker CD86 in the vicinity of granulomas. Furthermore, mDCs isolated from BAL displayed normal immune reactivity compared to healthy controls. Intriguingly, also in non-granulomatous mucosal airway biopsies from sarcoidosis patients there was a tendency towards increased numbers and maturation of mDCs, compared to healthy controls, although this was not statistically significant. It is tempting to speculate that these mucosal DCs are activated upon acquiring antigen from the airway lumen and subsequently present the antigen in a draining lymph node or are involved in mucosal granuloma formation. Taken together our results strongly support the notion that mDCs are involved in granuloma formation and maintenance in sarcoidosis, rather than the alternative that DCs are defective in supporting the adaptive immune system in antigen clearance. Our study included patients with newly diagnosed stage I/II sarcoidosis, we did not include patients with stage III/IV disease, and our results are therefore limited to patients presenting with early disease. We did not find a correlation between stage I or II and number of mDCs or pDCs. However, chest X-ray stage often poorly correlates with disease activity or progression. Thus, the role of DCs in granuloma formation or maintenance in subjects with advanced disease remains to be determined.

In conclusion, we provide evidence for the involvement of DCs in antigen presentation and granuloma formation at the site of disease in pulmonary sarcoidosis patients. Intrinsic genetic alterations in key APCs may underlie the exaggerated immune response to a hard to discern antigen that is characteristic of sarcoidosis. Immunological measurements and functional examination of DC and T cell subsets from large groups of carefully genotyped patients should prove very interesting.

## Competing interests

The authors declare that they have no competing interests.

## Authors' contributions

BL, HCH, BB and BtB designed the study design and the experiments. BtB, AK, FM and HH were responsible for flow cytometry and data collection. BtB, BB, BL, and RH analyzed the data. BtB, BB and RH drafted the manuscript. BtB, AK, FM, HH, HCH, RH, BL and BB read, critically revised and approved the final manuscript. All authors read and approved the final manuscript.
